# GIS and Injury Prevention and Control: History, Challenges, and Opportunities

**DOI:** 10.3390/ijerph7031002

**Published:** 2010-03-11

**Authors:** Nathaniel Bell, Nadine Schuurman

**Affiliations:** Department of Geography, Simon Fraser University, 8888 University Drive, Burnaby, British Columbia, V5A 1S6, Canada; E-Mail: nathaniel.bell@vch.ca

**Keywords:** injury prevention, geographic information systems (GIS), social determinants

## Abstract

Intentional and unintentional injury is the leading cause of death and potential years of life lost in the first four decades of life in industrialized countries around the world. Despite surgical innovations and improved access to emergency care, research has shown that certain populations remain particularly vulnerable to the risks and consequences of injury. Recent evidence has shown that the analytical, data linkage, and mapping tools of geographic information systems (GIS) technology provide can further address these determinants and identify populations in need. This paper traces the history of injury prevention and discusses current and future challenges in furthering our understanding of the determinants of injury through the use of GIS.

## 1. Introduction

Intentional and unintentional injury is the leading cause of death and potential years of life lost in the first four decades of life in industrialized countries around the world [1,2]. Critiques of contemporary injury prevention epidemiology have shown that despite improved access to healthcare services, intentional and unintentional injuries are strongly associated with relative disparities in socio-economic status (SES). However, the relationship is not universal. Socio-economic indicators are differentially related to age [3], gender [4], ethnicity [5], occupation [6], population density [7], and behaviour [8] and each of these characteristics interact differently according to the specific cause of trauma [9]. Researchers have also increasingly utilized geographic information systems (GIS) to better understand how the spatial organization of social and physical processes converge to either shelter or expose individuals to potentially harmful events [10–14]. This paper reviews core epidemiological and geographic contributions that have helped shaped our understanding of the social and physical determinants of injury and highlights theoretical and methodological approaches that have the capacity to increase our understanding of its environmental determinants. Context is provided from a Canadian injury prevention research perspective.

## 2. Perspectives toward Injury Prevention and Control

Injury has been defined as bodily lesions at the organic level, resulting from unintentional or intentional acute exposure to energy (mechanical, thermal, electrical, chemical or radiant) or the insufficiency of vital elements (e.g., oxygen) that exceed the threshold of physiological tolerance [15]. In order to prevent injury, strategies have focused on both its prevention and management, or control, to minimize its effect and optimize outcomes of an injury. Prevention can be targeted at both a population- and individual-scale; encompassing numerous strategies, techniques, or programs designed to eliminate or reduce its occurrence. Control efforts follow the traditional primary, secondary and tertiary disease prevention triad and are similarly aimed at minimizing the short- and long- term consequences of its effect.

### 2.1. Early Perspectives

In 1965, injuries in the USA accounted for over 52 million hospitalizations, resulted in 107,000 deaths and over 400,000 disabilities [16]. At the time, the state of critical care in the USA was so poor that military personnel returning from overseas military conflicts publicly asserted that if critically injured the odds of survival were better in the combat zone than on any city street in America [16]. Dr. William Haddon Jr., one of the foremost experts in injury prevention epidemiology, summarized then national and international perspectives toward injury prevention and was one of the first to develop an independent scientific field dedicated to its study [17]. Three distinct interrelated advancements in injury prevention and control evolved from this publication, including the emphasis on stronger scientific and research-based protocols, enacting legislation to reduce exposure to hazardous environments, and refining the coordination and delivery of emergency healthcare resources [18]. These initiatives have helped improve our ability to not only predict the occurrence of injury, but also better understand the environment in which injuries occur and dispatch the necessary emergency medical systems to improve outcomes [19–25].

Important as these developments might be, evidence has also shown that systems advances have not suppressed a growing societal health problem [26]. In fact, the disparity between what is known about the determinants of injury and what is done in terms of actually preventing it is greater than any other major health problem, including both HIV and AIDS [27]. As with other health conditions, alternative models of injury prevention have been underplayed in favour of the more predominant approach that equates better outcomes with improved access to healthcare services [28]. This research gap has also been attributed to barriers to data, resource limitations, a lack of generalizability of population-level indicators associated with incidence rates, as well as the presumption that factors such as social or economic position are not amenable to public health intervention [29,30].

### 2.2. Transitioning Perspectives

Beginning at least as early as the mid 1990’s, injury preventionists began utilizing research from behavioural science to identify particular aspects about human behaviour that either increased or decreased the effectiveness of traditionally more passive legislative and systems approach toward injury prevention [31,32]. In fire safety prevention, for example, smoke alarms were once considered a panacea for reducing burn and inhalation-related injuries. However, ongoing deaths and injury from residential fires have resulted in a growing recognition of the need for educational and behavioural change. Injury preventionists are now educating individuals to regularly test smoke alarm batteries and minimizing barriers for doing so (e.g., access to a step ladder), as well as pointing out the ineffectiveness of these programs if similar practices are not adhered to by neighbouring residents [26,33].

While this transition has helped to consolidate the strengths of passive prevention interventions within more active efforts of identifying how individuals interpret and approach ‘risk’, it remains problematic when educational and outreach programs are constructed independent of broader attention toward the individual’s social or physical environment. For example, burn/fire-related injury prevention efforts in Canada have primarily addressed risks that occur in the kitchen [34–37], from the misuse of cigarettes or alcohol [38], or resulting from improperly positioned/faulty electrical heaters and electrical wiring [39], while leaving largely underdeveloped any theoretical perspectives of why these risks may systematically vary among certain population groups.

Evidence derived from other health outcome studies has shown that key components thought to contribute to the effectiveness of a personal prevention program may be missed when efforts focus exclusively on ‘lifestyle’ choices measured through such risk modifiers as behavioural patterns [40]. Syme (1990), for example, found that nearly half of all persons selected for a risk factor intervention trial were unable to follow the recommendations for dietary change and smoking cessation [41]. One of the limitations posited from these findings was that in focusing exclusively on the individual, preventionists failed to acknowledge broader social and cultural forces that may have affected these outcomes, such as stress and empowerment disparities associated with employment hierarchies [28]. To place injuries within the context of broader social or economic conditions throughout society is necessary to identify whether factors external to the individual are useful and relevant contexts for explaining why certain populations are continually at a greater risk of injury.

### 2.3. Social Determinants of Injury

Some of the most compelling research on the relationship between health outcomes and variations in social and economic conditions is in reference to findings first published in the Report of the Working Group on Inequalities in Health and the Whitehall longitudinal studies of cardiovascular disease among British civil servants [42,43]. These and other evidence have shown that there is no threshold between status and health and that the widening gap in relative material wealth has led the vast majority of the populations—not just the poor—to disproportionately experience poorer health outcomes with each stepwise decrease in social position [44]. It is important to recognize that these findings emphasize relative mortality risk, not absolute risk. Death rates are decreasing for everyone in industrialized countries, but not at the same relative rate.

Pertaining to injury, Kim *et al.* (2007) raised a significant socio-economic and geographic question, “Why do places matter for injury risk?” [45]. Among children, for example, a recent study conducted by Edwards *et al.* (2006) found that children with unemployed parents were 13 times more likely to die from an injury as were children who lived in substantially more socially and economically privileged households [46]. At the individual scale, it was posited that the increased risk of injury potentially stemmed from psychosocial challenges associated with unemployment and its effects on parental supervision [46]. When ‘place’ is identified as representing a location, one can also point to influences of SES, as unemployment holds a direct link to community wealth and the ability to determine, in part, local access to healthcare services, procuring the means to pay for goods such as pedestrian traffic lights and safe playgrounds, as well as in increasing the ability to maintain strong patterns of residential stability that may indirectly lower crime [30]. Among youths, these factors become increasingly important as their ability to control their surroundings is quite limited [45]. If costs preclude areas from having playgrounds more children are likely to play in the street, abandoned buildings, or other hazardous areas, which all increase the likelihood for injury [47].

## 3. Mapping Place Effects on Injury

### 3.1. Measuring the Social Determinants of Injury

Measuring place effects on injury is similarly associated with an aspect of geographic scale. Quantifying this relationship also requires the use of a basic assumption that some defining ‘condition’ can be held constant over geographic space and over some span of time [48]. Most often, these two presumptions are imbedded in the reliance on national censuses as proxy representations of either individual-level or neighbourhood-level social and economic conditions. This follows a well-known interest in quantifying how relative variations in both physical and social aspects of places parallel variations in health outcomes [49,50].

Compositional models of this effect, for example, have been used to assess if relative variations in SES within one geographic area correspond with variations in the same area’s injury morbidity and mortality levels [51,52]. Compositional models are measured directly, through indicators such as average income, or indirectly, using either singular or aggregated indicators reflective of social, economic, and cultural status. In addition, injuries have been posited to vary according to the context of the social and physical environment where one lives independent of the strength of their own or that of their families social and economic position [8,30,53]. Multilevel models separately analyze the variance both between and within areal units so as to obtain a nested hierarchy of contextual as well as compositional influences on individual health outcomes [54–57]. These findings point to how the absence or unequal distribution of many aspects of ‘place’ interact with one’s individual circumstance (e.g., income, employment status) and may influence their health status [57,58].

It is important to recognize, however, that both composition and context affect how poverty and poorer living conditions may influence patterns or risk of injury, but data constraints often limit injury preventionists to studying incidence patterns of injury using aggregated socio-economic data taken from the census. This is troublesome because of the ecological fallacy, which occurs whenever a researcher makes assumptions about an individual based on aggregated data from a group of individuals [59]. Although multilevel models can circumvent the ecological fallacy they can be similarly criticized for overselling the meaningfulness of contextual effects on health that necessarily must be derived from proxy indicators [60]. These problems can be further compounded due to the level of representativeness in the data [61]. In Canada, for instance, the census is particularly poor in capturing meaningful socio-economic information among First Nations peoples living on reserves [62].

### 3.2. Mapping the Spatial Determinants of Injury

Geographic information systems (GIS) are computer information platforms designed to collect, manage, store, and analyze spatial and non-spatial data, as well as combine data sources to help describe the world around us [63,64]. GIS offer injury preventionists numerous sets of tools for understanding how the spatial organization of social and physical processes converge to either shelter or expose individuals to potentially harmful events. These might include the effects of neighbourhood socio-economic environments, accessibility to resources, municipal or regional zoning policies, and other artifacts from the public space such as the quality of parks and other recreation areas [65–68]. Perhaps most importantly, GIS allows researchers to observe how the amalgamation of spatial and non-spatial data sources yields important knowledge about social and structural processes that might not have been otherwise possible.

Within geographic disciplines, numerous attempts have been made to convey, spatially, that injury patterns can be investigated—and mapped—to better understand the environmental circumstances against which they occur. The earliest examples of this line of reasoning date back to at least the 1980’s. Whitelegg (1987) reflected on the significance of spatial patterns to help tease out the interrelationships between human behaviour, perception, scale and spatially varying susceptibility to hazards [69]. Similarly, Joly *et al.* (1991) used mapping to indicate concentrations of injuries and the utility of small-area census boundaries to illustrate how demographic structure and population density factors affected injury [70]. In fact, the continued collaboration between geographers and injury preventionists has fuelled a burgeoning interest in quantifying the influence of neighbourhood socioeconomic context on incidence patterns of injury [71–75].

Importantly, the increasing analytical power of GIS has enabled injury preventionists to evolve from simple a-spatial rate mapping techniques into more complex analysis of spatial interactions. For example, Lightstone’s [76] distance-based analysis of childhood pedestrian injuries in relation to street networks highlighted the physical relationship between proximity, transportation structures, and residential dwellings, highlighted by an incremental decrease in injury prevalence with increased distance between collision sites and residential dwellings [76]. This evidence has been used to fuel new perspectives toward traffic density, intersection design, or modifications to the built environment [76]. Parallel research has similarly been used to quantify the impact of roadway conditions, street geometries, and traffic control devices and incidence patterns of injury, particularly in and around alcohol outlet locations [14,66,77].

## 4. Theoretical Considerations

As important as the continued development of GIS for injury prevention might be, thus far its use has fit the traditionally more passive lens of injury prevention. This has included mapping aspects of environmental exposures [78,79], structuring legislative improvements [73], or measuring the effects of location and distances on the delivery of emergency medical care services [12,80]. In addition, descriptions of singular variables associated with increased risk of injury, such as ‘drunk driving’ and ‘speeding’ have been replaced by ‘location to alcohol facility’ and ‘distance to road network’ [14,66,76,81], which limits the creation of new evidence as to the graded relationship between status and health. In other health outcomes literature, GIS are emerging as key tools for corroborating evidence linking social and economic processes to population health outcomes [82–85]. Whilst the inclusion of GIS in mapping injuries are testaments to growing interest in recognizing its societal burden, increasing spatial inequalities require that researchers take a stronger role in building evidence of the parallel relationship between injury and social inequalities.

GIS are increasingly applied for assessing how both poverty and aspects from the built environment correspond with incidence patterns of injury. The following sections contextualize research techniques of particular interest for increasing our understanding of place effects on injury.

### 4.1. Working with Administrative Datasets

Our understanding of place effects on injury depends almost entirely on evidence derived from administrative datasets. In Canada for example, resource allocation formulas for monitoring injuries on aboriginal reserves are primarily derived from provincial and health region statistics, which are the largest of the health authority catchment units [86]. However, many other scales operate within these boundaries that may be better suited for identifying local variations in utilization or need of healthcare services by population sub-groups. For example, Mao *et al.* (1992) demonstrated that mortality concentrations on reserves are potentially more reflective of actual risk levels if the reference populations exclude major urban centres, which tend to downgrade small area rates in favour of the larger populations [87].

Mao *et al.*’s (1992) technique was a derivative of a probability map. Probability mapping techniques combine the strengths of classic rate mapping, but control for population variability by adjusting the significance of the population at risk using information taken from adjacent areas [88]. They are similar to a standard mortality ratio, but reveal the likelihood that the incidence rate would be significant if it were the same for the spatially adjacent reference population. This can help reduce bias from the small numbers problem, which arises due to the common reliance on census administrative geographies to map population aggregates at the finest scale possible while still having access to the descriptive attribute tables about the population [89].

**Figure 1.**
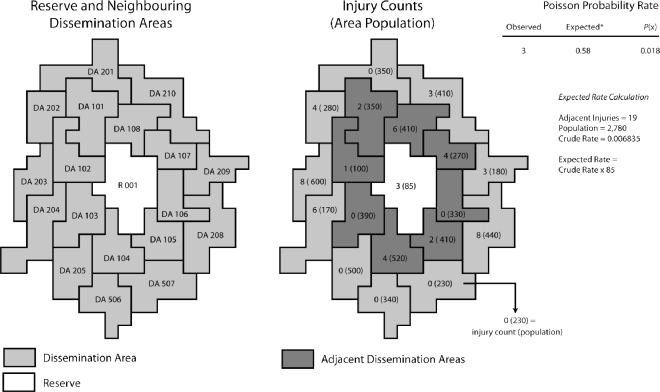
Adjacency model and Poisson probability calculation. The adjacency functions in GIS allow identification of adjacent DA’s that can be used to build reference ‘neighbourhoods’ when modeling incidence patterns of injuries among areas with low populations.

When mapped, probability techniques also offer a number of criteria for deriving more meaningful reference populations than are currently employed by provincial health authorities. For example, in contrast to referencing regional populations when addressing high or low risk incidence rates of injuries on aboriginal reserves, GIS could potentially be used to define each reserve’s “neighbourhood” according to the immediately adjacent communities. Figure 1 illustrates how Poisson mapping can be used to identify if incidence patterns of injuries in areas with few populations are significantly higher or lower than rates within the immediately surrounding areas. Such a technique can be used to investigate health outcomes on reserves relative to populations that are likely to be more socially, economically and geographically relative communities than the broader regional populations. For two cogent summaries of probability mapping techniques see [88,90].

Within Canada, provincial and aboriginal communities are moving toward a more local perspective of monitoring health outcomes, particularly among populations living on reserves [91,92]. Research has shown the important nuances in health outcomes among First Nation’s Peoples that is exposed when focusing more closely on communities [93]. This is an important research area and developing GIS-based approaches that are extensions of these perspectives can help redefine and facilitate a more spatialized understanding of local environments and the burden of injury.

### 4.2. Implications on Non-Independence

In many instances when an event’s significance is assessed as a product of its location additional care must also be given to the influence on the location itself in subsequent correlation analyses. Areas that are close together tend to have similar characteristics, or are said to be autocorrelated, which may confound etiological models of injury, as the assumption of variable independence cannot be sustained. A common approach to control for the distribution of events is to identify spatial autocorrelation [94,95].

The spatial autocorrelation statistic is similar to a traditional descriptive statistic such as the mean or the standard deviation, but it also reveals information about how events are arranged in space [94–96]. The utility of the statistic for injury surveillance is two-fold. First, quantifying the spatial variation of injuries allows researchers to infer the extent to which injury risk may be characterized by its location, independent of the inclusion of additional compositional or contextual variables [97]. For example, neighbouring areas tend to be more similar than dissimilar in terms of socio-economic or demographic factors. Spatial autocorrelation models also allow researchers to determine the likelihood that explanatory socio-economic factors are spatially independent, which is beneficial for identifying type I errors [14,66,77].

Thus far, injury preventionists have employed Moran’s I autocorrelation technique to uncover spatial patterning of injuries in relation to SES mechanisms [10,66,77,97]. However, Moran’s I is based on the assumption that the measured phenomenon (either SES or the health outcome) follows a Gaussian (e.g., normal curve) spatial process [98,99]. Unlike variations in SES, injuries, are decidedly non-normal events. Unfortunately, out-of-the-box analysis tools in many GIS software systems assume a normal distribution in the input data and there has been little discussion regarding these limitations in the analysis of health outcome data, particularly injuries [68].

### 4.3. The Modifiable Effect of Boundary Design

Problems associated with geographic scale and adjacency arise as a result of the dependence on aggregate data and the associated spatial boundaries. To date, injury prevention literature has focused on identifying ecological processes rather than evaluating, spatially, how different methodologies might redefine how we conceptualize this relationship. Statistical conclusions from aggregated data are susceptible to the magnitude of data aggregation and the ways in which the units are subdivided whenever researchers work with data that are partitioned by administrative fiat. This problem, more formally referred to as the *modifiable areal unit problem* (MAUP), has long been the focus of attempts to disentangle the statistical effects that arise out of various partitioning of areal datasets – especially those derived from the census [83,100,101].

**Figure 2.**
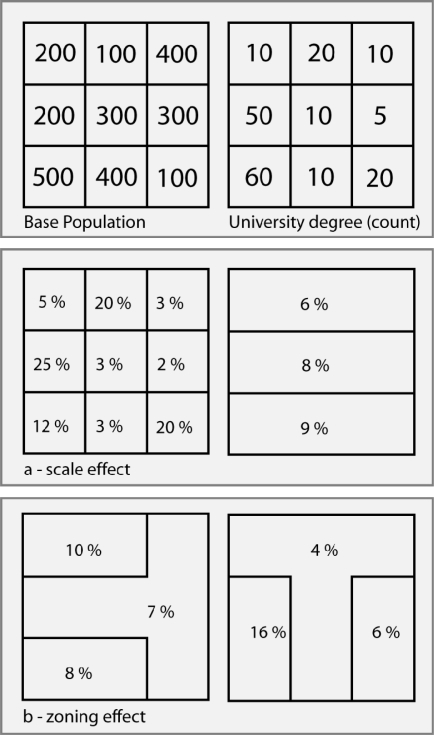
The scale and zoning effect of the modifiable areal unit problem (MAUP). Changes in either the scale or areal partitioning of the census units will bring about changes in the association between the independent and dependent variables. This is illustrated in Figure 2 using the proportion of population with a university degree as an example. Both subsets a and b illustrate how different permutations of the nine cells representing the numerator and denominator populations can alter the final statistic of university attainment percentages.

Attempts to address the MAUP are primarily condensed into two distinct, but closely related problems. The first is the well-known scale effect. As the name implies, different statistical results are obtained from the same set of geographic units when they are organized into an increasingly larger (or smaller) spatial scale [59]. Not unrelated, the zoning effect refers to the effect of basing a hypothesis from areal geographic units, which, if subdivided differently *at the same spatial extent*, may or may not lead the investigator to conclude differently [102]. Figure 2 illustrates these two problems. Recognition of the MAUP is of particular importance in ecological assessments of injuries as social and economic determinants of health may operate at different spatial extents [102–105]. However, explicit attention to its effects has yet to be addressed within the injury prevention literature. This is problematic as the influence of SES may have substantially different influences at both proximal and more distal geographic scales.

For example, targeting ‘high risk’ neighbourhoods where intentional injuries occur more frequently might be a suitable scale for the analysis of morbidity and mortality data, but we might also equally infer that this epidemic is a reflection of society, thus suggesting that comparisons are more accurate if individual risk patterns are contextualized against larger municipal or regional environments. The versatility of GIS enables the analysis of variation across multiple spatial extents. However, this is not an entirely satisfactory solution as this does not allow us to determine if incidence patterns are an artefact of how the areal units are partitioned. Researchers have rarely moved beyond the manipulation of geographic units defined by the census to model neighbourhood influences on health—thus failing to address the extent that place effects on health are linked to the way in which the data are aggregated.

## 5. Conclusion

Injury remains a hidden epidemic and its social determinants should remain a concern among researchers engaged in healthcare policy and health promotion. Injury preventionists today find themselves in a unique position for refining our understanding of contemporary research into health and well-being, particularly injuries, as space and place might be considered intrinsic characteristics of injury—a health condition whose cause originates from outside the body.

Research on the social determinants of injuries is still emerging, and is much enriched when also explored using geographic information technology. One of integral benefits of GIS is that it often builds on top of traditional analytic methods whilst recognizing that events are also likely to be spatially linked. Geographical concepts can be used to understand the complexities of our social environment and help preventionists better understand why some populations consistently and persistently experience greater risks of injury more than others. However, at the intersection of this interdisciplinary merger there is a need to continue to identify how the information-intensive analysis associated with GIS can be used to corroborate the growing evidence in favour of investigating health outcomes at the local, community scale, and in conjunction with multiple and interrelated social, economic, and environmental indicators. This collaboration constitutes an important component of modern public health research into injury surveillance and prevention.

To date, however, this research intersection has primarily utilized GIS for identifying ecological processes associated with increased risk. There has been little attention directed toward the sensitivity of ecological models to variation that arises out of the reliance on administrative data. Researchers in injury prevention must remain vigilant of the dynamics as well as the artefact of administrative datasets. Using GIS, nearly any data from a health registry can be encoded with geographic identifiers and explored, spatially, to uncover patterns in morbidity and mortality in ways that were previously either not possible or only feasible at a national scale. GIS is potentially a powerful tool for elucidating and communicating injury trends and the technology can offer both confirmatory and exploratory data solutions to a variety of questions related to its occurrence. The research intersection between GIS and injury prevention and control is still being developed and there is much potential for the technology to serve as a means of analysis and communication of health trends and their graded nature.
